# The characteristics of gut microbiota in colorectal cancer with different microsatellite states

**DOI:** 10.3389/fcimb.2026.1729343

**Published:** 2026-07-08

**Authors:** Yao Yao, Ren Bingbing, Shan Junqi, Wang Hao, Li Xinyu, Sun Yanlai

**Affiliations:** 1 Department 2 of Colorectal Surgery, Shandong Cancer Hospital and Institute, Shandong First Medical University and Shandong Academy of Medical Sciences, Jinan, China; 2 Department of Bone and Soft Surgery, Shandong Cancer Hospital and Institute, Shandong First Medical University and Shandong Academy of Medical Sciences, Jinan, China; 3 Department of Pediatric Surgery, Tianjin Medical University General Hospital, Tianjin, China; 4 Department of General Surgery, Shandong Provincial Public Health Clinical Center, Jinan, China; 5 Yongkang Orthopaedic Hospital, Yongkang Sixth People's Hospital, Jinhua, Zhejiang, China

**Keywords:** akkermansia, colorectal cancer, faecalibactrium, gut microbiota, immunotherapy, microsatellites

## Abstract

**Background:**

The efficacy of immunotherapy varies between MSI-H and MSS colorectal cancer(CRC) patients. Multiple studies have shown that gut microbiota is closely related to CRC, but the causality of them has yet to be fully established. This study aims to look for differential bacteria and related pathways by collecting feces from patients with different MS of CRC and healthy individuals, providing direction for immunotherapy of MSS CRC patients.

**Objective:**

1. Explore the differences in gut microbiota between MSI-H and MSS CRC. 2.Provide new direction for the future clinical treatment of MSS CRC patients and improve their prognosis.

**Methods:**

From October 2021 to December 2025, fecal samples and medical records were collected from healthy individuals and patients with different genotypes of MSS and MSI-H CRC admitted to Shandong Cancer Hospital and Institute. 28 fecal samples were collected from MSI-H, MSS CRC patients and healthy individuals respectively. Then 84 fecal samples were analyzed using 16S rDNA sequencing. Finally the differential microbial communities in the fecal samples of MSI-H and MSS CRC patients were analyzed based on the sequencing results, and healthy human fecal samples were used as controls to explore the role of differential microbial communities in CRC immunotherapy.

**Result:**

The collected 84 fecal samples were subjected to 16s rDNA sequencing analysis. Through Venn plot analysis, it was found that there are 1774 same bacterial species in MSS and MSI-H CRC patients, 3951 unique bacterial species in MSS CRC patients, and 2571 unique bacterial species in MSI-H CRC patients. The species richness of intestinal microbiota in MSS was higher than that in MSI-H CRC patients. Through species analysis, differential bacteria were identified among the samples submitted for testing, and further validation was obtained. Finally, based on the test results and comprehensive analysis of relevant literature, this study found that the bacteria with the most significant difference at phylum level between MSI-H and MSS CRC patients are Bacillota(p=0.024), Bacteroidota(p=0.024), Pseudomonadota(p=0.024), Actinomycetota(p=0.026), Verrucomicrobiota(p=0.037), and at genus level are Akkermansia(p=0.022), Alistipes(p=0.032), Anaerostipes(p=0.034), ASF356(p=0.016), Bacteroides(p=0.032), Bilophila(p=0.007), Coprococcus(p=0.037), Culicoidibacter(p=0.031), Enterocloster(p=0.049), Escherichia-Shigella(p=0.045), Faecalibacterium(0.042), Flavobacterium(p=0.037), Mediterraneibacter(p=0.037), Monoglobus(p=0.028), Oscillibacter(p=0.024), Parabacteroides(p=0.038), Ruthenibacterium(p=0.041), Streptococcus(p=0.015), Terrisporobacter(p=0.019), Tyzzerella(p=0.045), UCG-002(p=0.025), UCG-005(p=0.027), UCG-009(p=0.032), Veillonella(p=0.020). Among them, the relationship between Akkermansia, Faecalibactrium, Coprococcus and Bacteroides with immunotherapy is a hot research topic at home and abroad.

**Conclusion:**

There are differences in the species composition of gut microbiota between MSI-H and MSS CRC patients, which may provide new directions for further research.

## Introduction

Colorectal cancer (CRC) is a significant global health concern, which ranked third in cancer incidence and second in mortality worldwide in 2020 (American Cancer Society, 2024). According to the World Health Organization (WHO), more than 1.9 million new cases and more than 930,000 deaths due to CRC were estimated to have occurred worldwide in 2020 (Morgan et al., 2022). Likewise, the incidence rate and mortality of CRC also stay in a high position without going down in China. The latest statistical data from the China National Cancer Center shows that among common malignant tumors in China, colorectal cancer ranks second in terms of morbidity and mortality in 2022 (Han et al., 2024). CRC has served as a genetic and biological paradigm for the evolution of solid tumors (Li et al., 2021). According to the differences in genomes, there are two theories classifying the etiology of colorectal cancer into chromosomal instability and microsatellite instability (Faghfuri and Gholizadeh, 2024). Chromosomal instability accounts for 80% of the pathogenesis of colorectal cancer. This pathway is caused by mutations in oncogenes and tumor suppressor genes, which interact through multiple pathways and factors, ultimately leading to the occurrence of colorectal cancer (Ai et al., 2011). Microsatellite (MS) is a short repetitive sequence in DNA, and microsatellite instability (MSI) is characterized by a high quantity of mutations in microsatellite locations which results from mutations or silencing of genes coding for mismatch repair (MMR) proteins (MLH1, MSH2, MSH6 and PMS2). According to the different states of MS, CRC can be divided into microsatellite instability-high (MSI-H) subtype, Microsatellite instability-low(MSI-L) subtype and microsatellite stability (MSS) subtype (Evrard et al., 2019). Approximately, in MSI-H subtype colorectal cancer, hereditary non polypic colorectal cancer (HNPCC), also known as Lynch syndrome, accounts for 85-90%, while sporadic colorectal cancer accounts for 10-15% (Ai et al., 2011). In colorectal cancer especially for MSI-H subtype patients, the application of immunotherapy has become an important treatment strategy. Treatment methods of immunotherapy mainly include immune checkpoint inhibitors, vaccine therapy, CAR-T cell therapy and combination immunotherapy. However, between the two subtypes of CRC, due to the differences in tumor microenvironment (TME), there exists a significant discrepancy in the effectiveness of immunotherapy. MSS CRC has the characteristic of cold tumor, manifested by reduced levels of tumor immune cell infiltration and tumor mutational burden (TMB) (Chen and Mellman, 2017). In the TME of cold tumor, the large proportion of cancer-related fibroblasts enhances the tumor matrix barrier function, hindering the efficacy of immune drug (Wu and Dai, 2017). Therefore, cold tumor is often difficult to be recognized and cleared by the immune system due to a lack of sufficient immune cell intervention and immune response. At present, patients with cold tumor, including MSS CRC, mainly improve their prognosis through treatment regimens such as chemotherapy combined with targeted therapy or radiotherapy (Yang et al., 2018). MSI-H CRC is usually characterized by early onset, commonly seen in the right colon, and higher immune cell infiltration. It differs from MSS CRC in biological and clinical features. Microsatellite instability high (MSI-H) cancers encompass a subset of colorectal cancers sensitive to immune checkpoint inhibitors (ICIs) (Heregger et al., 2023). MSI-H CRC exhibit low tumor differentiation, accompanied by mucinous histological features, abundant lymphocyte infiltration in TME (Kim et al., 1994), and high PD-L1 expression levels, indicating that MSI-H CRC patients can benefit more from PD-1 inhibitors and improve prognosis (Narayanan et al., 2019).

CRC is characterized as a multifactorial and heterogeneous disease. Related studies have shown that gut microbiota greatly affects the occurrence and development of colorectal cancer (Wong and Yu, 2019). Compared with germ free (GF) rats, conventional rats have a higher susceptibility to intestinal tumors (Laqueur et al., 1967). At present, there are few reports on the differences in gut microbiota among patients with different genotypes of colorectal cancer. Jin (Jin et al., 2022)found that there are differences between the deficient DNA mismatch repair (dMMR) group and proficient DNA mismatch repair (pMMR) group in cancer tissues, specifically manifested in the proportion of major microbiota and differences at the phylum level between the two groups. Compared with the pMMR group, Firmicutes, Actinobacteria, Fusobacteria, and Verrucomicobia were significantly enriched in the dMMR group, but the abundance of Proteobacteria was reduced. In addition, CRC with different gene state has different gut microbiota metabolic function, which may further affect the human physiological function. This may indicate that potential mechanism related to gut microbiota is involved in the occurrence and development of different gene state CRC.

In this study, to investigate the differences in CRCs’ gut microbiota between MSS and MSI-H subtype, we collected 600 fecal samples including normal individuals, CRC patients with MSS and MSI-H subtype. The MS status of CRC patients were confirmed via the results of tumor biopsy tissue gene testing. Finally, 28 samples of MSI-H colorectal cancer patients were obtained, 28 MSS CRC and 28 normal human fecal samples were matched with similar basic information (including gender, age, BMI, tumor location, tumor stage, etc.). Then, we evaluated and compared the differences in gut microbiota richness and composition between different MS states’ fecal samples through 16S ribosomal DNA (rDNA) sequencing analysis.

## Materials and methods

### 1. Subject enrollment and sample preparation

**Figure 1:**
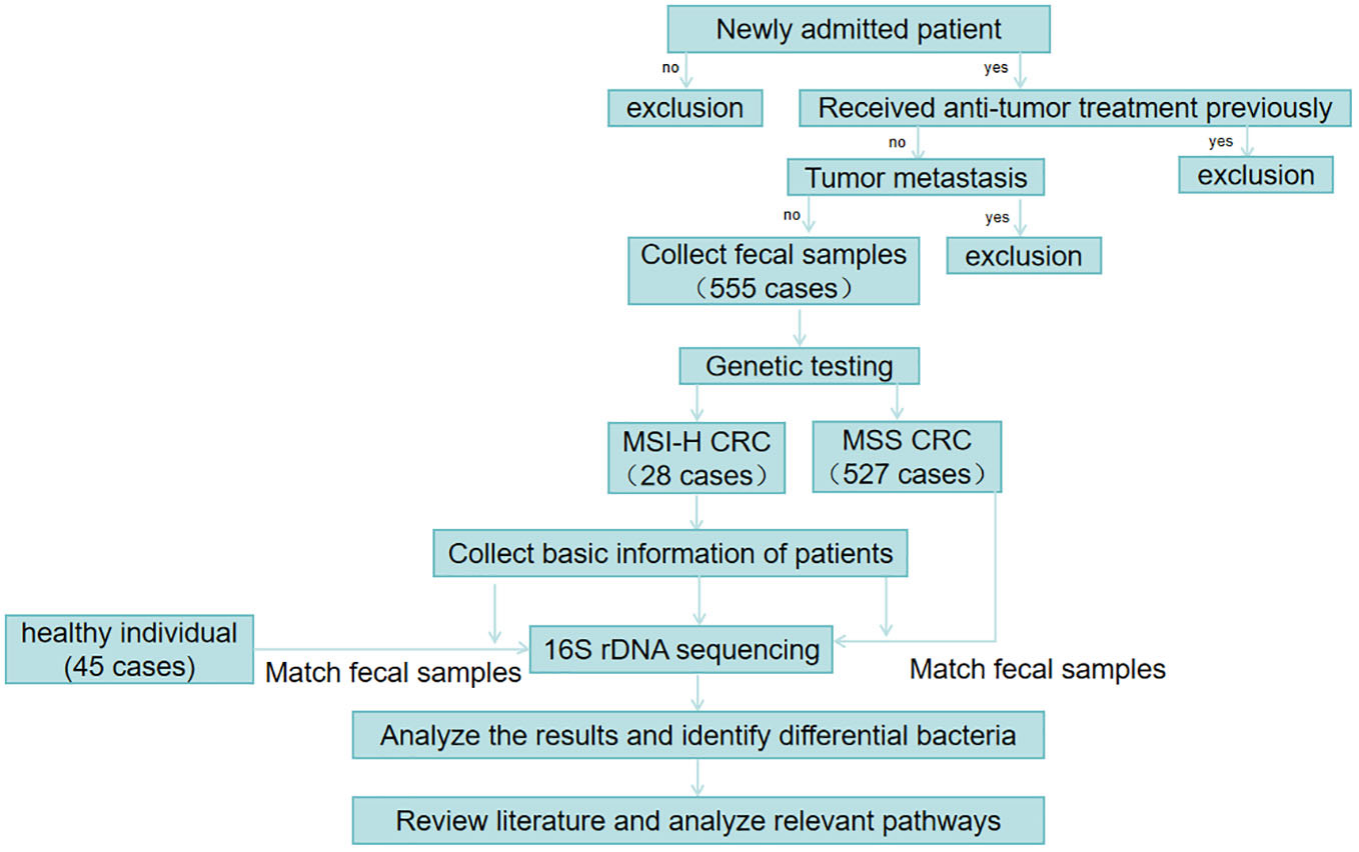
Flow chart of experimental design.

As shown in **Figure 1**, this study enrolled 555 initial diagnosis CRC patients and 45 healthy person from October 01, 2021 to December 31, 2025 at the Department 2 of Colorectal Surgery, Shandong Cancer Hospital and Institute. For patients with CRC, the inclusion criteria were (1) Confirmed by colonoscopy and biopsy pathology as a patient with colorectal adenocarcinoma; (2) Patients with CRC who have not received treatment at the initial diagnosis; The exclusion criteria were (1) Imaging confirmed of patients with distant metastasis of CRC; (2) Patients who have received the following treatments: radiation therapy, chemotherapy, immunotherapy, targeted drug therapy, surgical resection, endoscopic mucosal dissection (EMD) or endoscopic mucosal resection (EMR) and others; (3) Patients with primary disease combined with malignant tumors of other organs; (4) Secondary cancer patients; (5) Patients who have taken antibiotics in the past month; (6) Patients who have recently lived in extreme environments; (7) Patients with long-term day night inversion or insomnia; (8) All patients who have received other treatment options for CRC not mentioned above; (9) All unmentioned patients who had extreme lifestyle. For healthy individuals, the inclusion criteria was that individuals were not found systemic tumors, gastrointestinal adenomas or polyps after health examination. The exclusion criteria were (1) Healthy individuals who have taken antibiotics in the past month; (2) Healthy individuals who have recently lived in extreme environments; (3) Healthy individuals with long-term day night inversion or insomnia; (4) Healthy individuals with extreme lifestyles not mentioned above. The study was conducted according to the guidelines of the Declaration of Helsinki, and approved by the Ethics Committee of Shandong Cancer Hospital and Institute. Written informed consent was obtained from all subjects involved in the study.

According to the inclusion and exclusion criteria, we collected stool samples from CRC patients and healthy person according to the following methods. When healthy person or CRC patients (on the first day of admission and without using any medication) defecate at any time, they will be instructed to put the disposable sterile container on the toilet after urinating. Then defecate into the disposable sterile container and put the appropriate amount of stools into sterile stool collection tubes (≥6g per tube) with wearing the disposable sterile gloves. Finally, we will retrieve the fecal sample after completing the collection as soon as possible. The total number of collected CRC’s fecal samples was 555, healthy individuals’ fecal samples was 45.

The CRC patients included in this study were queried for tumor biopsy tissue gene testing results through the system in Shandong Cancer Hospital and Institute, and their microsatellite status was recorded. 28(28/555,5.05%) MSI-H CRC and 527(527/555, 94.96%) MSS CRC patients were ultimately screened out. Collect basic information of the 28 MSI-H CRC patients mentioned above, including gender, age, BMI, medical history and so on. Match the fecal samples from 527 MSS type CRC patients and 45 healthy individuals based on their basic information. Finally, 28 MSS CRC patients and 28 healthy persons with similar basic information to 28 MSI-H CRC patients were selected for the next experiment. Transfer the fecal samples to the testing site as soon as possible for 16s rDNA sequencing analysis based on the cold chain.

### 2. 16S rDNA sequencing

#### 2.1 DNA extractions, PCR amplification and 16S rDNA sequencing

DNA from different samples was extracted using the CTAB according to manufacturer’s instructions. Nuclear-free water was used for blank. The total DNA was eluted in 50μL of Elution buffer and stored at -80°C until measurement in the PCR. The PCR products were purified by AMPure XT beads (Beckman Coulter Genomics, Danvers, MA, USA) and quantified by Qubit(Invitrogen, USA). The amplicon pools were prepared for sequencing and the size and quantity of the amplicon library were assessed on Agilent 2100 Bioanalyzer (Agilent, USA) and with the Library Quantification Kit for Illumina (Kapa Biosciences, Woburn, MA, USA), respectively. The libraries were sequenced on NovaSeq PE250 platform.

#### 2.2 Data analysis

The basic information of samples was analyzed using SPSS software(29.0.2.0). Samples were sequenced on an Illumina NovaSeq platform. Paired-end reads were merged using FLASH. Quality filtering on the raw reads were performed under specific filtering conditions to obtain the high-quality clean tags according to the fqtrim(v0.94). Chimeric sequences were filtered using Vsearch software(v2.3.4). After dereplication using DADA2,we obtained feature table and feature sequence. Then according to SILVA(release 138) classifier, feature abundance was normalized using relative abundance of each sample. Alpha and Beta diversity were calculated by QIIME2,the graphs were drew by R package.

## Results

### 1. Basic information of samples

**Figure 2:**
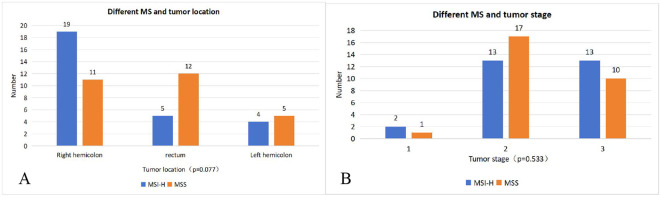
Different MS with tumor location and stage. **(A)** Different MS and tumor location, **(B)** Different MS and tumor stage.

The basic information of samples was analyzed using SPSS software(29.0.2.0). A total of 84 samples were enrolled in this study, with an average age of (52.72 ± 17.208) years. The average age of MSS group is (64.50 ± 9.442) years, MSI-H group is (53.79 ± 13.357) years, and healthy person group is (39.93 ± 18.108) years. There is no statistically significant difference in the age of samples in this three groups (p=0.072), as well as between two groups of tumor patients (p = 0.570). Among the 84 enrolled samples, 49 cases are male (49/84, 58.3%) and 35 cases are female (35/84, 41.7%). There is no significant difference in gender among the three groups (p = 0.822), as well as between two groups of tumor patients (p = 0.589). As shown in **Figure 2**, there is no statistical difference in tumor location and stage between the two groups of CRC patients.

### 2. Venn diagram

**Figure 3:**
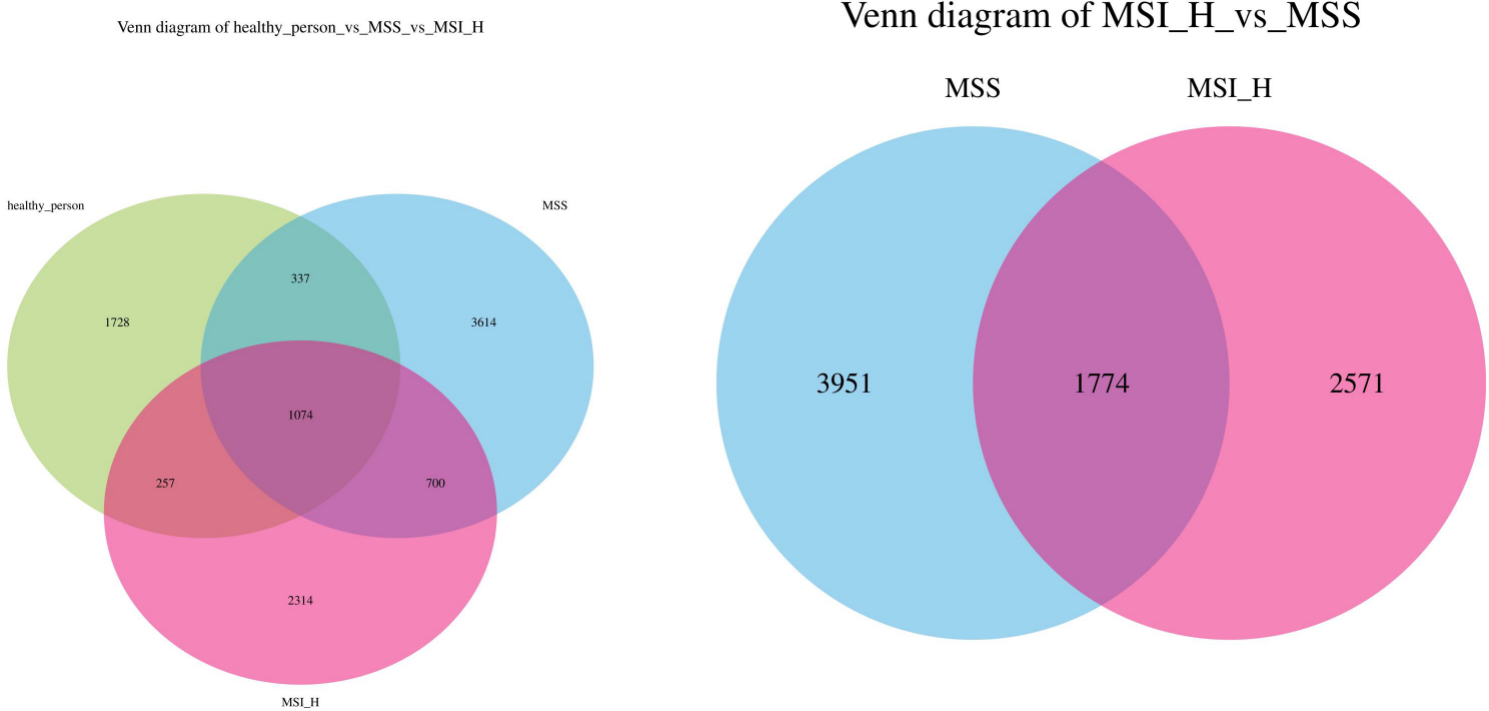
Venn diagram.

The Venn diagram can provide a more intuitive representation of the number of common and unique Amplitude Sequence Variants among three groups (Amplitude Sequence Variants, also known as ASV abundance tables, refer to unique sequences obtained through sequencing and sequence processing, typically used to represent different species or strains in microbial communities). According to the Venn diagram (**Figure 3**), there are 1774 bacterial species jointly owned in the group of MSS and MSI-H CRC patients, 5725 bacterial species in the group of MSS CRC patients, 4345 bacterial species in the group of MSI-H CRC patients, and 3450 bacterial species in the group of healthy persons. There is no statistical difference in the abundance of intestinal flora among the three groups (p = 0.199), as well as between two groups of tumor patients (p = 0.157).

### 3. Alpha and Beta diversity

Alpha and Beta diversity constitute the overall diversity or biological heterogeneity of a certain environmental community.

**Figure 4:**
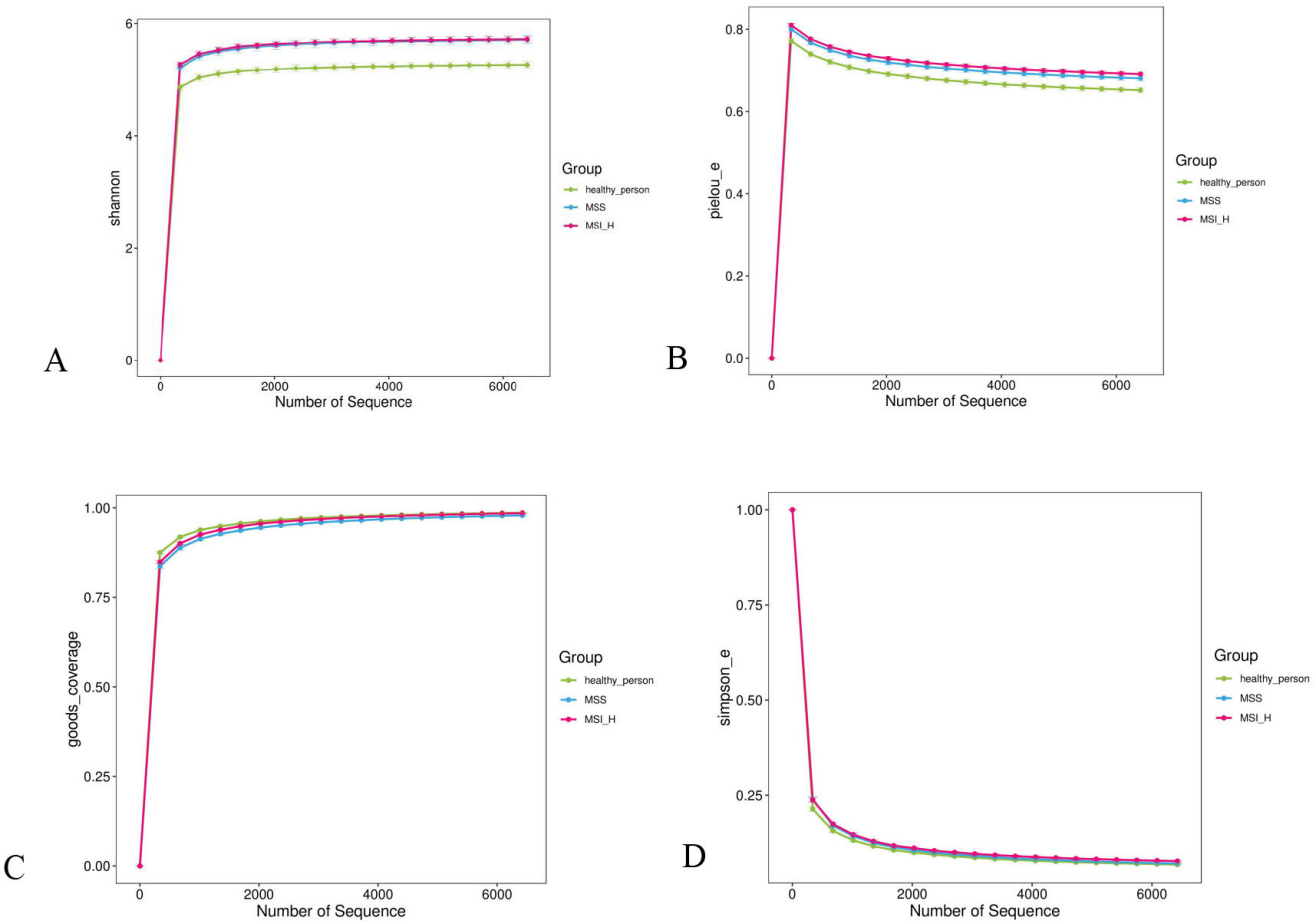
Alpha diversity. **(A)** shannon, **(B)** pielou-e, **(C)** good- coverage, **(D)** simpson-e.

Alpha diversity refers to the diversity within a specific environment or ecosystem, which reflects species richness, evenness, and sequencing depth. They are mainly reflected through indices such as Chao1, Goods coverage, Observed species, Pielou, Shannon and Simpson. The dilution curve can reflect the abundance of gut microbiota in feces. When the curve tends to flatten, it indicates that the sequencing data volume is gradually reasonable. In **Figure 4**, the alpha diversity curves of three groups tend to flatten out, indicating the rationality of the samples.

Beta diversity refers to the species differences between different environmental communities. Its analysis usually starts with calculating the distance matrix between environmental samples, which includes the distance between any two samples. It is mainly observed through below methods such as Principal Component Analysis (PCA), Principal Coordinates Analysis (PCoA), Multidimensional Scaling Analysis (NMDS) and Clustering Analysis (UPGMA) to observe the differences between samples.

**Figure 5:**
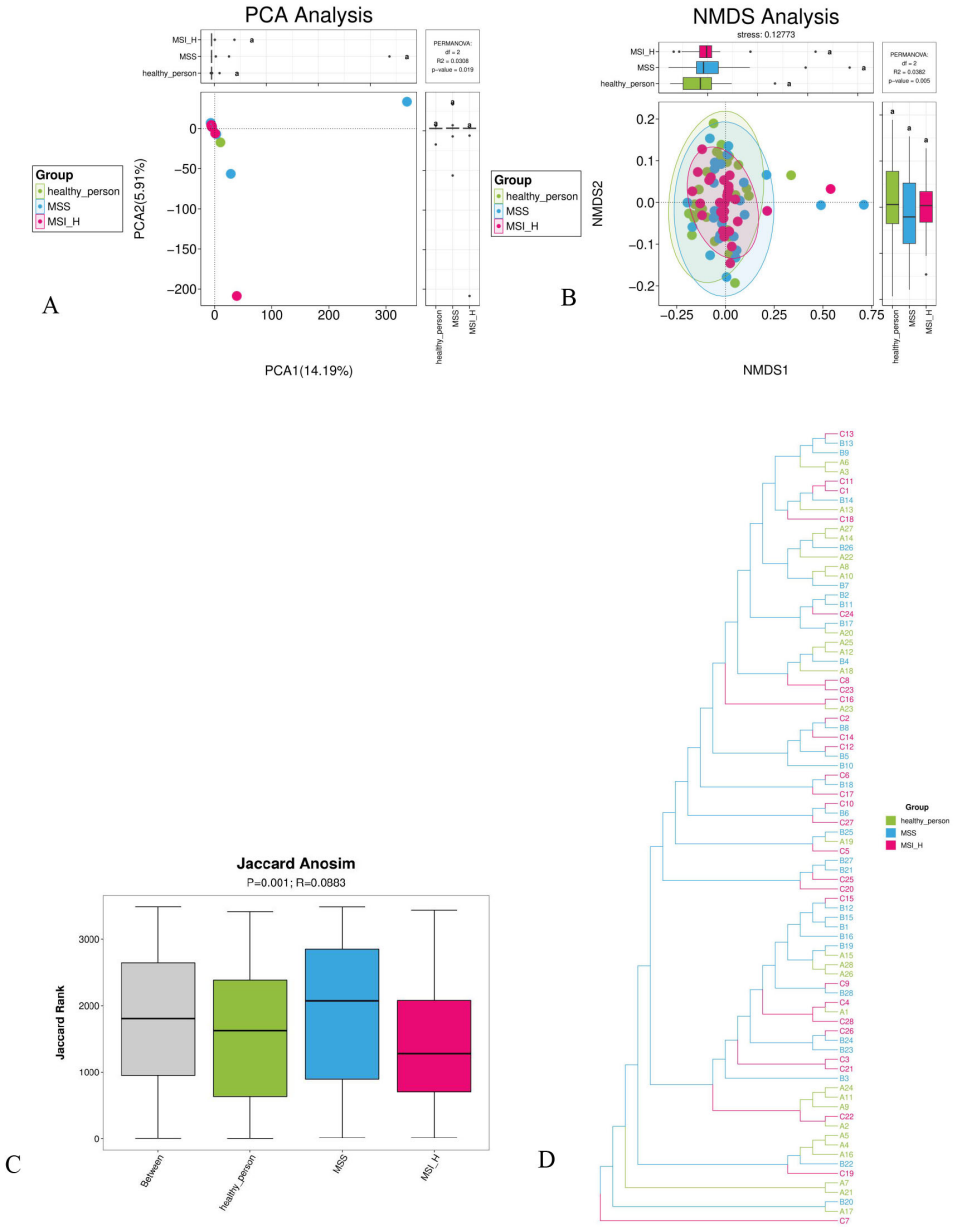
Beta diversity. **(A)** PCA, **(B)** NMDS, **(C)** UPGMA, **(D)** Anosim.

**Figure 5A** is PCA, Different colors in the figure represent different groups, and dots of the same color represent different samples of the same group. Each group is indicated by an elliptical area with a 95% confidence interval. The distance between samples reflects the difference degree of their microbial composition and structure - the closer the distance is, the smaller the difference is; The further the distance, the greater the difference. The upper right corner of the figure shows the results of the difference analysis of the three groups of bacteria, p=0.019, which indicates that the difference is statistically significant and reliable.

**Figure 5B** shows NMDS. Each point in the figure represents a sample, and the color represents different groups. The distance between samples represents the degree of difference in their community structure. P = 0.005 is shown in the upper right corner, indicating that there are differences in the flora of three samples.

**Figure 5C** shows UPGMA. Analysis shows that the branches of the clustering tree have varying lengths, indicating the differences in species between each sample. However, the specific species differences of three groups cannot be determined from this figure.

**Figure 5D** shows analysis of similarities (Anosim). Anosim is used to test whether the difference between groups is significantly greater than that within groups, so as to judge whether grouping is meaningful. As shown in the figure, p=0.001, R = 0.0883, indicates the significance of the differences between the three sample groups.

The above is the β diversity analysis of intestinal flora in healthy person, MSS and MSI-H CRC patients. However, analyzing the intestinal flora of MSS and MSI-H CRC patients, reveals no difference in β diversity. It indicates that compared with healthy person, the intestinal flora of CRC patients has changed. However, there is no significant difference in intestinal flora between CRC patients with different MS. In the following, we will analyze the differences of intestinal flora in CRC patients with MSS and MSI-H genotypes from different levels.

### 4. Species analysis

By analyzing the basic information of samples, Venn diagram, Alpha and Beta diversity, we have preliminarily understood the situation of the three groups of samples, as well as made clear the randomness of enrollment, the rationality of sequencing data volume and the credibility of the results. In order to clarify the differences of intestinal flora in CRC patients with different MS, we applied SPSS software to analyze the richness of flora. Due to the small or uneven data distribution, compared with Pearson chi square test, likelihood can more accurately reflect the true situation of the data. Species analysis is presented through column stacked chart, heatmaps and cluster diagram, analyzing the species in two groups of fecal samples at different levels of boundary, phylum, class, order, family, genus, and species. Due to the extensive research conducted at home and abroad on the level of phylum and genus, the analysis of these results will focus on the level of phylum and genus.

**Figure 6:**
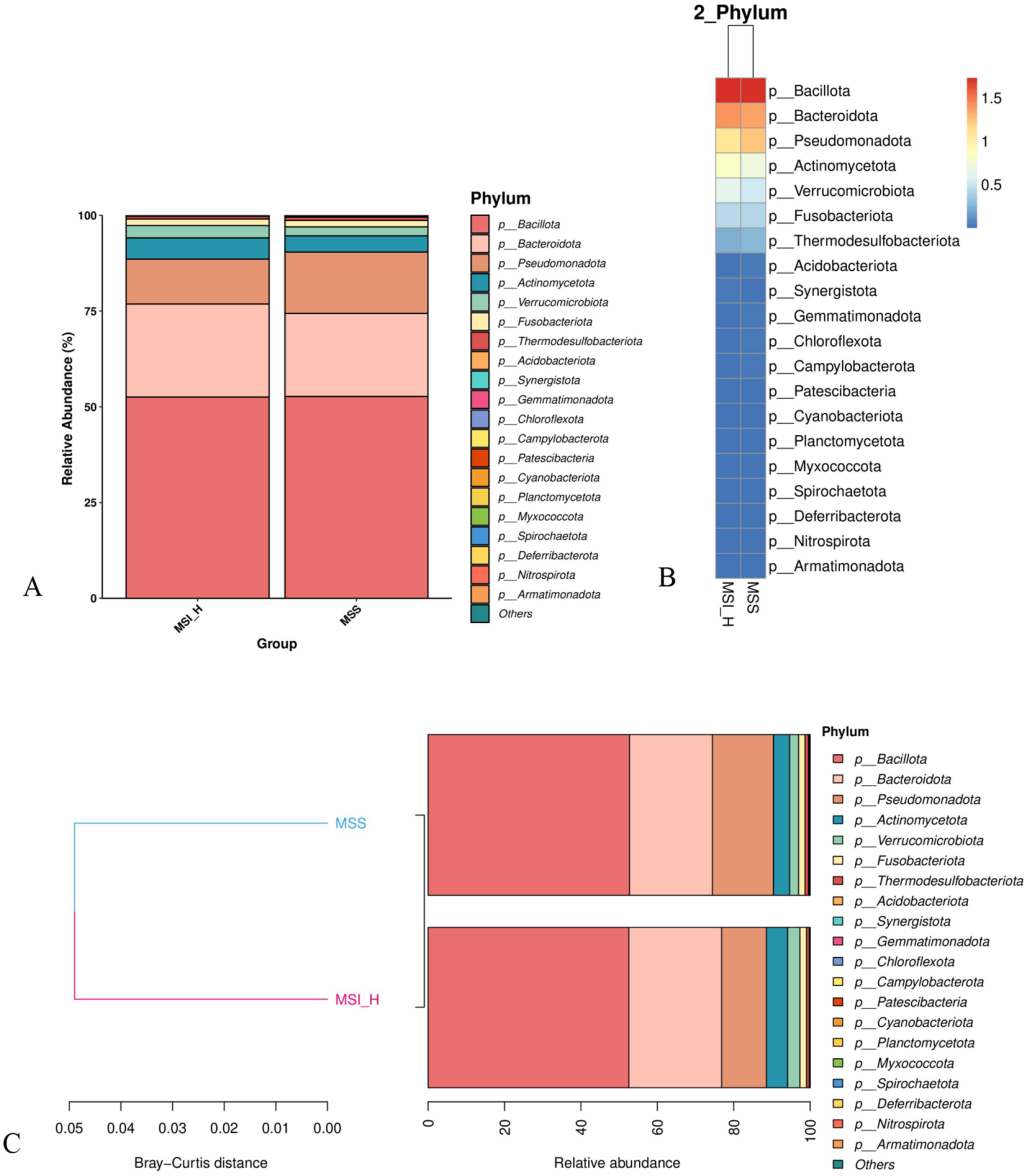
Differential flora at phylum level. **(A)** Stacked bar, **(B)** Heatmap, **(C)** Cluster.

**Figure 7:**
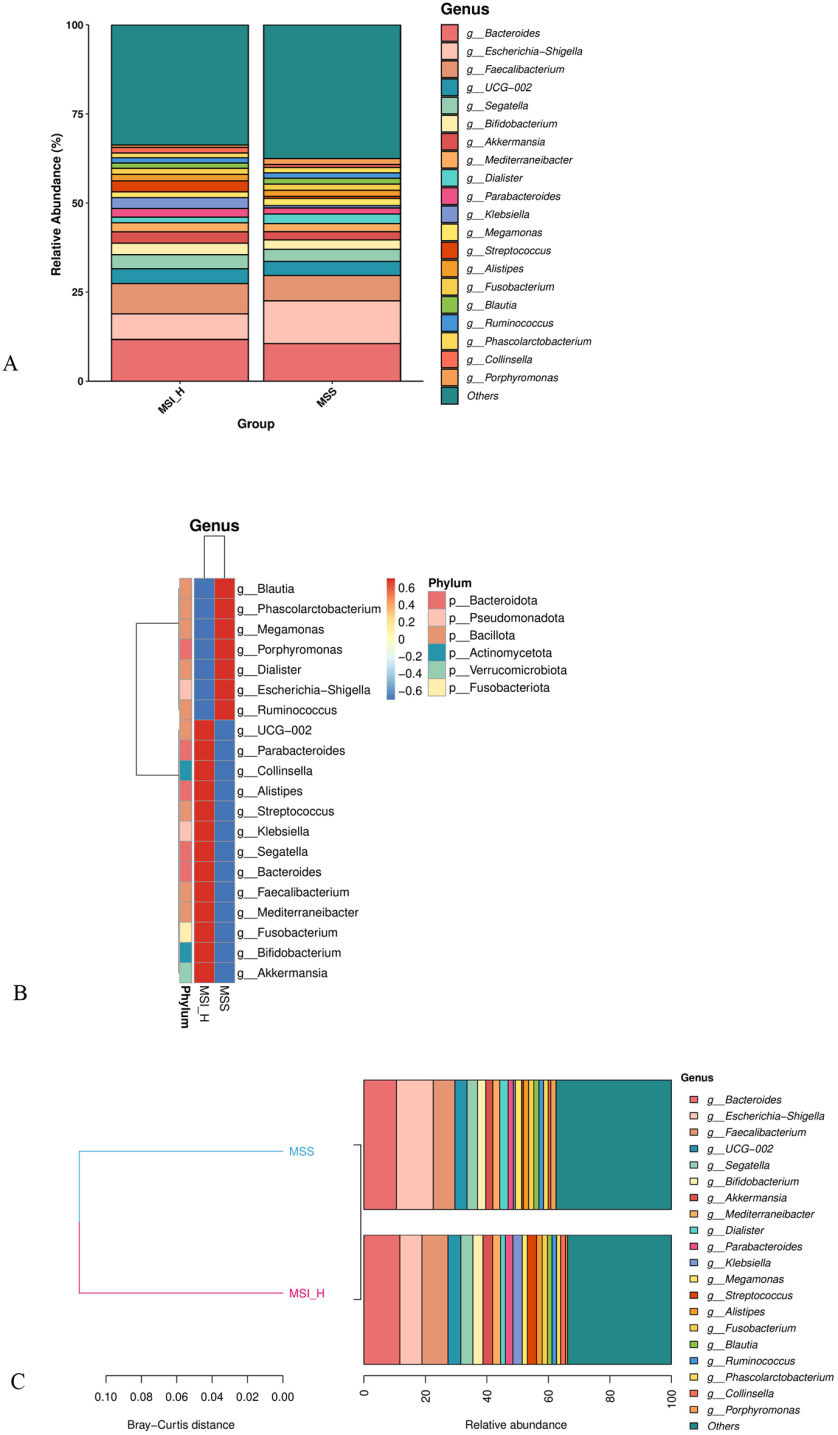
Differential flora at genus level. **(A)** Stacked bar, **(B)** Heatmap, **(C)** Cluster.

By analyzing the raw data, result shows that, in phylum level, the differential bacteria of MSS and MSI-H CRC patients are Bacillota(p=0.024), Bacteroidota(p=0.024), Pseudomonadota(p=0.024), Actinomycetota(p=0.026), Verrucomicrobiota(p=0.037). In genus level, the differential bacteria of MSS and MSI-H CRC patients are Akkermansia(p=0.022), Alistipes(p=0.032), Anaerostipes(p=0.034), ASF356(p=0.016), Bacteroides(p=0.032), Bilophila(p=0.007), Coprococcus(p=0.037), Culicoidibacter(p=0.031), Enterocloster(p=0.049), Escherichia-Shigella(p=0.045), Faecalibacterium(0.042), Flavobacterium(p=0.037), Mediterraneibacter(p=0.037), Monoglobus(p=0.028), Oscillibacter(p=0.024), Parabacteroides(p=0.038), Ruthenibacterium(p=0.041), Streptococcus(p=0.015), Terrisporobacter(p=0.019), Tyzzerella(p=0.045), UCG-002(p=0.025), UCG-005(p=0.027), UCG-009(p=0.032), Veillonella(p=0.020). To visually show the calculation results, we draw **Figures 6**, **7**. Column stacked chart is a commonly used data visualization chart, typically used to compare the proportion of different categories of data in the total amount. Heatmap is used to display the numerical or relative size of data through the depth of colors. Cluster diagram is used to display the clustering structure between samples or features in data.

## Discussion

Our body is a complicated complex, which consists of own cells and other microbiota (such as bacteria, archaea, viruses, fungi, etc.), which is known as the human microbiome (Kunz et al., 2009). Studies have shown that the occurrence of osteoporosis (OP), inflammatory bowel disease (IBD), CRC and other diseases are closely related to the dysbiosis of intestinal flora (Lynch and Pedersen, 2016; Bahney et al., 2019; Li et al., 2022). Intestinal flora plays an important role in the formation of the human immune system, inflammatory response, and physiological characteristics of tumors (Brennan and Garrett, 2016; Marchesi et al., 2016). The occurrence and development of CRC are affected by many factors, among which, the intestinal flora is related to DNA damage, inflammation regulation, oxidative stress and other tumor formation processes (Wong and Yu, 2019). In order to verify the influence of intestinal bacteria on CRC susceptibility, several scientific research teams conducted relevant research and found that the treated conventional rats had higher intestinal tumor susceptibility than germ free rats (Laqueur et al., 1967; Reddy et al., 1975). The abundance of beneficial bacteria such as Bifidobacterium and Streptococcus decreased in the intestine of patients with colorectal cancer. Enterococcus, Escherichia coli, Shigella, Klebsiella, Streptococcus and Peptostreptococcus increased, as well as the abundance of butyrate producing bacteria decreased significantly (Chattopadhyay et al., 2021; Liu et al., 2021). At present, the relevant mechanism of CRC caused by intestinal bacteria is not clear, which may be related to bacteria mediated DNA damage, inflammatory response, immune system abnormalities and abnormal cell behavior (Alhinai et al., 2019). Human intestinal flora is affected by many factors, including age, dietary habits, lifestyle, antibiotic use, environmental factors, etc (De Vrieze, 2013; Zhao et al., 2018; Guittar et al., 2019; Mortaş et al., 2022).

**Figure 8:**
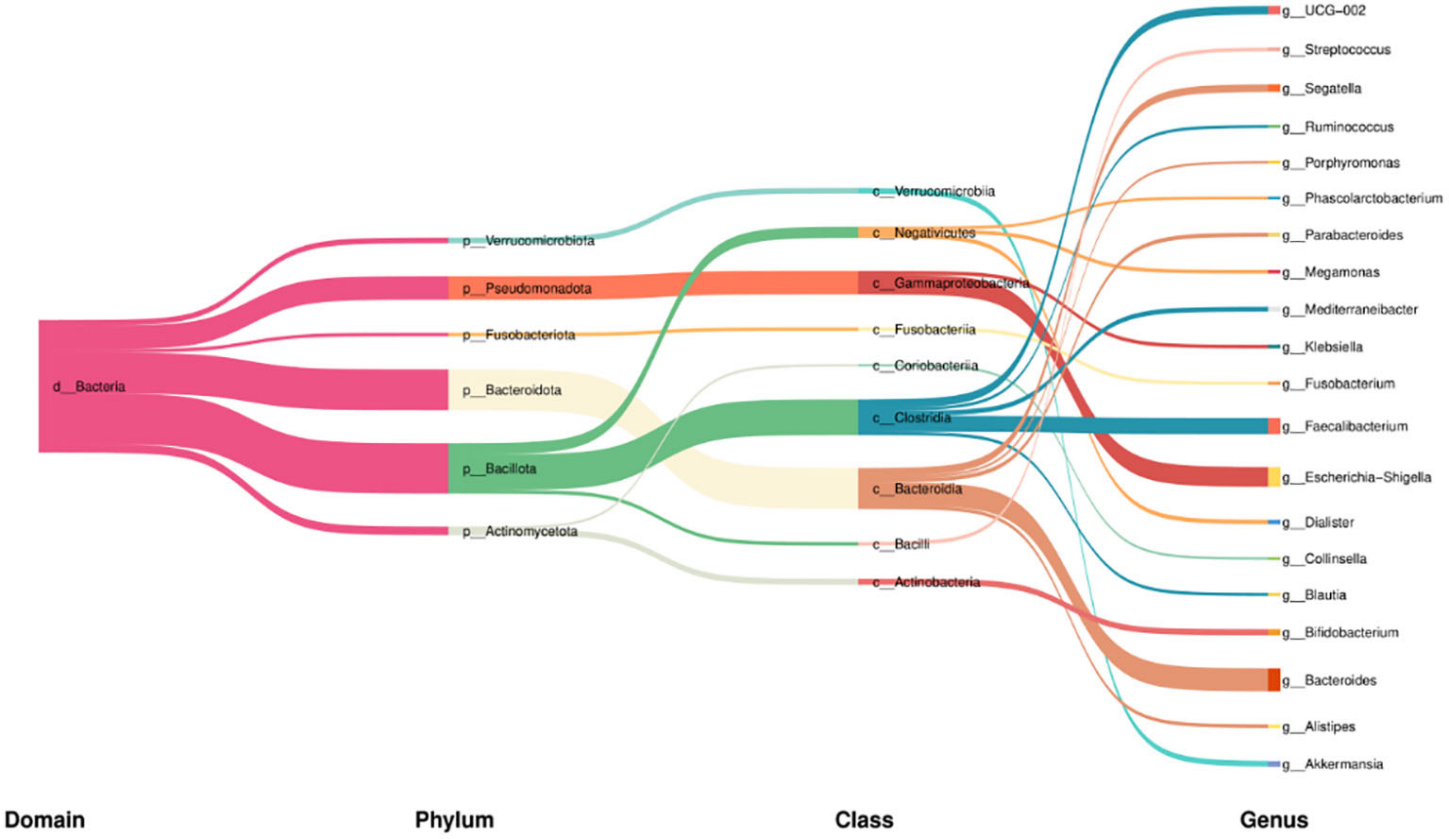
Sankey plots.

Therefore, the gut microbiota may be beneficial in the diagnosis and treatment of CRC; some bacteria may serve as biomarkers while others as regulators of chemotherapy and immunotherapy. Furthermore, metabolites produced by the gut microbiota play essential roles in the crosstalk with CRC cells (Qu et al., 2023). At the same time, Multiple studies both domestically and internationally have shown that MSI-H CRC patients can benefit more from immunotherapy compared to MSS CRC patients (Narayanan et al., 2019). This study conducted 16s rDNA sequencing on fecal samples of CRC patients with different genotypes, and according to the experimental results, the different flora of the two groups were found. At phylum level, the differential bacteria of MSS and MSI-H CRC patients are Bacillota, Bacteroidota, Pseudomonadota, Actinomycetota, Verrucomicrobiota. At genus level, the differential bacteria of MSS and MSI-H CRC patients are Akkermansia, Alistipes, Anaerostipes, ASF356, Bacteroides, Bilophila, Coprococcus, Culicoidibacter, Enterocloster, Escherichia-Shigella, Faecalibacterium, Flavobacterium, Mediterraneibacter, Monoglobus, Oscillibacter, Parabacteroides, Ruthenibacterium, Streptococcus, Terrisporobacter, Tyzzerella, UCG-002, UCG-005, UCG-009, Veillonella. **Figure 8** is Sankey plots, which is used to show the “flow” changes of data, mainly reflecting the proportion of species at the phylum to genus levels between different samples/groups. It can show: (1) the relative abundance of the flora of top20 genera in different groups/samples; (2) the proportion of the phyla to which these genus belong.

At phylum level, according to correlation literatures, Bacillota (also known as Firmicutes) and Bacteroidota are the two main forces of the intestinal flora. The ratio of the two phyla (F/B ratio) and the overall bacterial diversity can better predict the effect of immunotherapy than the level of a single phylum. The higher the diversity, the better the treatment effect. The F/B ratio is recognized for its crucial role in maintaining gut health and its association with different disorders, including IBD, obesity and diabetes (Yin et al., 2025). The F/B ratio of healthy person is usually between 0.5 and 1, indicating that the flora is in balance. If the ratio is high (for example, greater than 1), it is usually closely related to metabolic diseases such as obesity and diabetes. A lower F/B ratio is associated with young age, a lean phenotype, a balanced immune system and poor cardiovascular health, which are generally considered beneficial for overall health (Andoh et al., 2016; Koliada et al., 2017). Our result shows that the F/B of CRC patients with MSI-H (2.16) is lower than that of MSS (2.43). Through F/B we can guess that MSI-H CRC patients have a more balanced immune system and better immune response than MSS. This is also consistent with the fact. However, differences in study design, sample size, methods, study population and geographical location may all affect the results. Therefore, it is important to evaluate the relevant literature to comprehensively understand the gut microbiome (Yin et al., 2025).

At genus level, Akkermansia and Faecalibactrium are currently recognized by the medical community as key bacterial genera positively correlated with the efficacy of immunotherapy, particularly PD-1/PD-L1 inhibitors. In addition, Coprococcus and Bacteroides are also related to immune therapy or adverse reactions. Their relationship with immunotherapy will be analyzed below.

### 1. Akkermansia

Akkermansia (myxophilic Akkermansia,or AKK) is a probiotic that has gained significant attention in the fields of microbiology and medical research in recent years. This type of bacteria belongs to the gut microbiota and mainly grows on the surface of the intestinal mucosa in humans and other animals. Studies have shown that increasing the quantity of AKK bacteria can promote the health of intestinal mucosal, regulate the immune system, and reduce inflammatory reactions. Although AKK bacteria only account for 3% of the gut microbiota, current research has shown that their abundance is negatively correlated with the risk of various chronic diseases. For example, in patients with obesity, type 2 diabetes, inflammatory bowel disease, Alzheimer disease, Parkinson disease, CRC and other diseases, the number of AKK bacteria is descending, so the imbalance of intestinal flora may be closely related to the pathogenesis of the above diseases (Cani and De Vos, 2017; Vogt et al., 2017; Depommier et al., 2019; Ou et al., 2020; Qian et al., 2020; Ling et al., 2021; Cani et al., 2022).

There is a positive correlation between the abundance of AKK bacteria in the gut microbiome and the efficacy of immunotherapy for non-small cell lung cancer using PD-1/PD-L1 inhibitors (Derosa et al., 2022). Research has found that the high abundance of AKK bacteria in the gut is associated with the immune system’s more effective recognition and elimination of cancer cells, thereby enhancing the therapeutic effect of PD-1/PD-L1 inhibitors (Yu et al., 2017). AKK bacteria not only affect intestinal health, but may also become a potential target for improving the efficacy of immunotherapy. And it is expected to become the next generation of probiotics with clinical application prospects. However, the mechanism of interaction between AKK bacteria and human body remains unclear. Previous studies have found that AKK bacteria can regulate the energy balance and nutrient absorption process in the intestine, thereby affecting the metabolic processes of the whole body (Everard et al., 2013; Greer et al., 2016). Scholars have conducted in-depth research on the role of AKK bacteria in immune regulation through a series of technical means (Bae et al., 2022). They found that AKK bacteria can affect the function of human immune cells through phosphatidylethanolamine (PE) on their cell membrane, especially in promoting the secretion of specific cytokines. These phospholipid molecules can bind to receptors on the surface of immune cells, activating and inducing them to secrete key immune regulatory factors such as tumor necrosis factor alpha and interleukin-10. Specific PE variants, such as a15:0-i15:0 PE, contain methyl groups on both fatty acid chains, which are located on the 12th and 13th carbon atoms of each fatty acid chain. This special configuration of methylation may affect the biological activity and intercellular signaling ability of PE. PE molecules carrying methylated fatty acid chains may have specific interactions with other biomolecules, which can alter their behavior in the cell membrane, such as interactions with proteins or regulation of cellular signaling pathways. In addition, methylated fatty acids may affect membrane fluidity and cell responsiveness, further impacting the physiological and pathological state of cells. These characteristics make a15:0-i15:0 PE an important research object, which may play a key role in regulating cellular function and treating related diseases (Henke et al., 2019). However, the existing findings were obtained *in vitro* cell culture, and the role of AKK bacteria and their phospholipids *in vivo* remains to be studied, especially in pharmacological studies of a15:0-i15:0 PE.

Goldszmid and his colleagues found that a high fiber diet is beneficial for the colonization of gut microbiota such as Akkermansia. These gut microbiota induce the generation of macrophages with anti-cancer effects through the STING-IFN-I pathway, and increase the number as well as activity of natural killer cells, monocytes, and dendritic cells, thereby improving the tumor microenvironment and enhancing the efficacy of immunotherapy. Researchers have found that the gut microbiota under a high fiber diet can utilize the STING-IFN-I pathway to improve the tumor microenvironment, induce an increase in IFN-I levels, promote the expression of IFN-b1 in monocytes, and increase the expression of chemokine 1 in natural killer cells; After conducting relevant experiments on GF mice and mice with specific microbiota colonization, it was found that only the tumors of mice with Akkermansia microbiota colonization showed a significant increase in dendritic cells and a significant decrease in the proportion of macrophages that promote/inhibit cancer; After analyzing the intestines of normal mice, Akk only colonized mice, and GF mice, it was found that Akkermansia bacteria can produce all three factors that activate the STING pathway (cdAMP, cdGMP, cGAMP), and mainly produce cdAMP. Therefore, it can be considered that the Akkermansia microbiota is the most closely related to mononuclear phagocytes in tumors, mainly regulating mononuclear phagocytes in the tumor microenvironment by producing cdAMP. Finally, the above team also evaluated the efficacy of high fiber diet assisted immunotherapy and found that under high fiber diet, gut microbiota activates the STING-IFN-I pathway, improves the tumor microenvironment, and enhances the effectiveness of immunotherapy in mice (Lam et al., 2021).

### 2. Faecalibactrium and Coprococcus

Faecalibacterium is an important member of the Firmicutes phylum and is one of the frequent bacteria in the normal human microbiota. Faecalibacterium’s anti−inflammatory properties are widely known. Both of them can produce butyrate salts, which exerts anti-inflammatory, anti-tumor, and gut microbiota-regulating effects, making it a key biomarker for evaluating intestinal health (Miquel et al., 2013; Lopez-Siles et al., 2017; Lin et al., 2025). Butyrate metabolism is closely associated with immunotherapy (Bachem et al., 2025; Zhang et al., 2025). Butyrate promotes the stemness differentiation of CD8^+^ T cells by activating the transcription factor FOXO1. This increases the population of CD127^+^ CD8^+^ T cells with memory potential, enhances their anti-tumor activity and long-term proliferative capacity, and thereby improves the efficacy of immune checkpoint inhibitors such as anti-PD-1. In CRC, butyrate reduces PD-1 expression on the surface of CD8^+^ T cells, inhibits T cell exhaustion, restores their cytotoxic function, and promotes tumor cell killing. Furthermore, butyrate is capable of modulating the tumor immune microenvironment. It remodels the tumor microenvironment by epigenetically activating the CXCL11 enhancer (e.g., through increased histone acetylation) and recruiting the STAT4 transcription factor. This promotes CXCL11 secretion, which chemotactically recruits natural killer (NK) cells to infiltrate tumor tissues, thereby enhancing the anti-tumor immune response. In tumors such as hepatocellular carcinoma, butyrate inhibits tumor cell proliferation and reduces Ki67 expression, while simultaneously increasing NK cell infiltration and suppressing tumor growth. inally, butyrate helps maintain immune homeostasis. It can inhibit the NF-κB signaling pathway via the GPR109A receptor, reducing the release of pro-inflammatory cytokines (such as TNF-α and IL-6) and promoting the production of anti-inflammatory cytokines (such as IL-10). This regulates the Treg/Th17 cell balance and alleviates inflammatory responses, creating favorable conditions for immunotherapy.

### 3. Bacteroides

On May 30, 2025, a multi-institutional research team including the Broad Institute of MIT and Harvard published a paper online in Cell Host & Microbe (Brown et al., 2025), which reached the following conclusions: Sphingolipids (e.g., CerPE) carried by Bacteroides outer membrane vesicles OMVs induce anti-inflammatory responses by activating the host mevalonate pathway (a key route for cholesterol synthesis). Mechanistically, after OMV-delivered sphingolipids enter host cells, they upregulate genes for rate-limiting enzymes in the mevalonate pathway (such as Hmgcr and Hmgcs1), promoting IL-10 transcription and inhibiting IL-1β secretion; conversely, inhibiting this pathway blocks IL-10 production. *In vitro* experiments showed that supplementing sphingolipid-deficient OMVs with CerPE restored their anti-inflammatory function. *In vivo* experiments demonstrated that colonization with wild-type Bacteroides allowed sphingolipids to persist in intestinal tissues and activate the pathway, an effect not observed with sphingolipid-deficient strains. This research reveals a “sphingolipid-mevalonate-IL-10” anti-inflammatory axis, providing the first proof that microbial sphingolipids mediate immune homeostasis by regulating host metabolic pathways. It offers a new therapeutic direction for inflammatory diseases (such as enteritis) by targeting gut microbiota metabolites—for instance, promoting anti-inflammatory responses by enhancing sphingolipid-mevalonate pathway interactions—and lays the foundation for developing novel therapies based on microbial outer membrane vesicles.

## Deficiencies and prospects

This experiment analyses the differences in gut microbiota between MSS and MSI-H CRC patients through multi-level analysis. Although we have made some progress in this study, there are still some limitations. Firstly, larger sample size experiments are needed to improve the reliability and generalizability of research statistical results. Secondly, establishing corresponding basic experiments is the foundation for ensuring the reliability of experimental results. Our research team will continue to conduct in-depth research in this direction in future research plans.

Although domestic and foreign researchers have extensively explored the differences in gut microbiota through multidimensional and multi-level methods, and the relationship between gut microbiota and various diseases has gradually become clear, research on the differences in gut microbiota between MSS and MSI-H genotypes in CRC patients is still limited. To further explore the role and potential therapeutic value of gut microbiota in CRC of different genotypes, we will conduct larger scale clinical trials and in-depth basic experiments. Our team will also design relevant studies to explore whether gut bacteria are effective in treating CRC. In summary, through systematic and multidimensional research methods, we can not only verify the hypothesis of the relationship between gut microbiota and CRC, but also explore its feasibility as a potential therapeutic target, bringing hope and new treatment opportunities to CRC patients.

## Conclusion

There are differences in the species composition of gut microbiota between MSI-H and MSS CRC patients, which may provide new directions for further research.

## Data Availability

Raw data have been deposited to National Center for Biotechnology Information (NCBI) under the BioProject number PRJNA1484467.
